# The atherogenic index of plasma and the risk of mortality in incident dialysis patients: Results from a nationwide prospective cohort in Korea

**DOI:** 10.1371/journal.pone.0177499

**Published:** 2017-05-26

**Authors:** Mi Jung Lee, Jung Tak Park, Seung Hyeok Han, Yong-Lim Kim, Yon Su Kim, Chul Woo Yang, Nam-Ho Kim, Shin-Wook Kang, Hyung Jong Kim, Tae-Hyun Yoo

**Affiliations:** 1 Department of Internal Medicine, CHA Bundang Medical Center, CHA University, Seongnam, Korea; 2 Department of Internal Medicine, Yonsei University College of Medicine, Seoul, Korea; 3 Department of Internal Medicine, Kyungpook National University School of Medicine, Daegu, Korea; 4 Clinical Research Center for End-Stage Renal Disease, Daegu, Korea; 5 Department of Internal Medicine, Seoul National University College of Medicine, Seoul, Korea; 6 Department of Internal Medicine, Catholic University of Korea College of Medicine, Seoul, Korea; 7 Department of Internal Medicine, Chonnam National University Medical School, Gwangju, Korea; The University of Tokyo, JAPAN

## Abstract

**Background:**

The atherogenic index of plasma (AIP), which is the logarithmic ratio of triglyceride (TG) to high-density lipoprotein cholesterol (HDL-C), had a linear relationship with clinical outcomes in the general population. However, the association of each lipid profile, TG and HDL-C, with survival was not straightforward in dialysis patients. This non-linear association led us to further investigate the prognostic impact of the AIP in these patients.

**Methods:**

From a nationwide prospective cohort, 1,174 incident dialysis patients were included. Patients were categorized into quintiles according to the AIP. An independent association of the AIP with all-cause and cardiovascular mortality was determined.

**Results:**

During a mean follow-up duration of 33.2 months, 170 patients (14.5%) died, and cardiovascular death was observed in 55 patients (4.7%). Multivariate Cox analyses revealed that the lowest (quintile 1, hazard ratio [HR] = 1.76, 95% confidence interval [CI] = 1.02–3.03) and the highest (quintile 5, HR = 2.15, 95% CI = 1.26–3.65) AIP groups were significantly associated with higher all-cause mortality compared to patients in quintile 3 (reference group). In terms of cardiovascular mortality, only the highest AIP group (quintile 5, HR = 2.59, 95% CI = 1.06–6.34) was significantly associated with increased risk of mortality. Sensitivity analyses showed that a U-shaped association between the AIP and all-cause mortality remained significant in non-diabetic and underweight to normal body mass index patients.

**Conclusions:**

Both the highest and the lowest AIP groups were independently associated with all-cause mortality, showing a U-shaped association. It suggested further studies are needed to identify targets and subgroups that can benefit from intervention of the AIP in incident dialysis patients.

## Introduction

Dyslipidemia is an established risk factor for cardiovascular disease in the general population [1]. In particular, atherogenic dyslipidemia, which means a concurrence of high triglyceride (TG) and low high-density lipoprotein cholesterol (HDL-C) levels, has been shown to be closely associated with adverse clinical outcomes in the general population [2], as well as high risk patients [3–6]. Gaziano et al. [2] first explored the combining effect of elevated TG and low HDL-C and demonstrated that the TG to HDL-C ratio was a strong predictor of myocardial infarction in the general population. Moreover, a greater TG/HDL-C ratio was implicated with fatal or non-fatal cardiovascular disease [3–6] and all-cause mortality [3, 6] in women with high cardiovascular risk [3], obese type 2 diabetes patients [4], patients with prior stroke or transient ischemic attack receiving statin therapy [5], and essential hypertension patients [6]. The atherogenic index of plasma (AIP), which is the logarithmic ratio of TG to HDL-C, was also found to be correlated with cardiovascular risk [7]. The close association of the AIP and cardiovascular risk has been mainly explained by lipoprotein particle size [7], insulin resistance [8–10], and metabolic syndrome [11], all of which are important risk factors for cardiovascular disease. To date, two studies have investigated the association between the TG/HDL-C ratio and clinical outcomes in end-stage renal disease (ESRD) patients treated by dialysis therapy [12, 13]. These studies indicated that the highest quintile or quartile of the TG/HDL-C ratio was associated with increased risk of cardiovascular and all-cause mortality compared with the lowest part [12, 13]. However, several previous studies showed that the prognostic impact of each lipid profile, TG and HDL-C, was not straightforward in dialysis patients [14–17]. In hemodialysis (HD) patients, the association between TG and mortality showed a U-shaped association [15], whereas a direct association of TG with mortality was observed in peritoneal dialysis (PD) patients [14]. In terms of HDL-C, there have been discordant results from no association [15, 17] to U-shaped relationship [16]. Furthermore, the retrospective nature of previous studies [13] and limited data of a single center study [12, 13] led us to further investigate the prognostic impact of the AIP in our cohort. Therefore, in the present study, the association of the AIP with all-cause and cardiovascular mortality was evaluated in an incident dialysis population from a nationwide prospective observational multicenter cohort.

## Materials and methods

### Study participants

**Fig 1:**
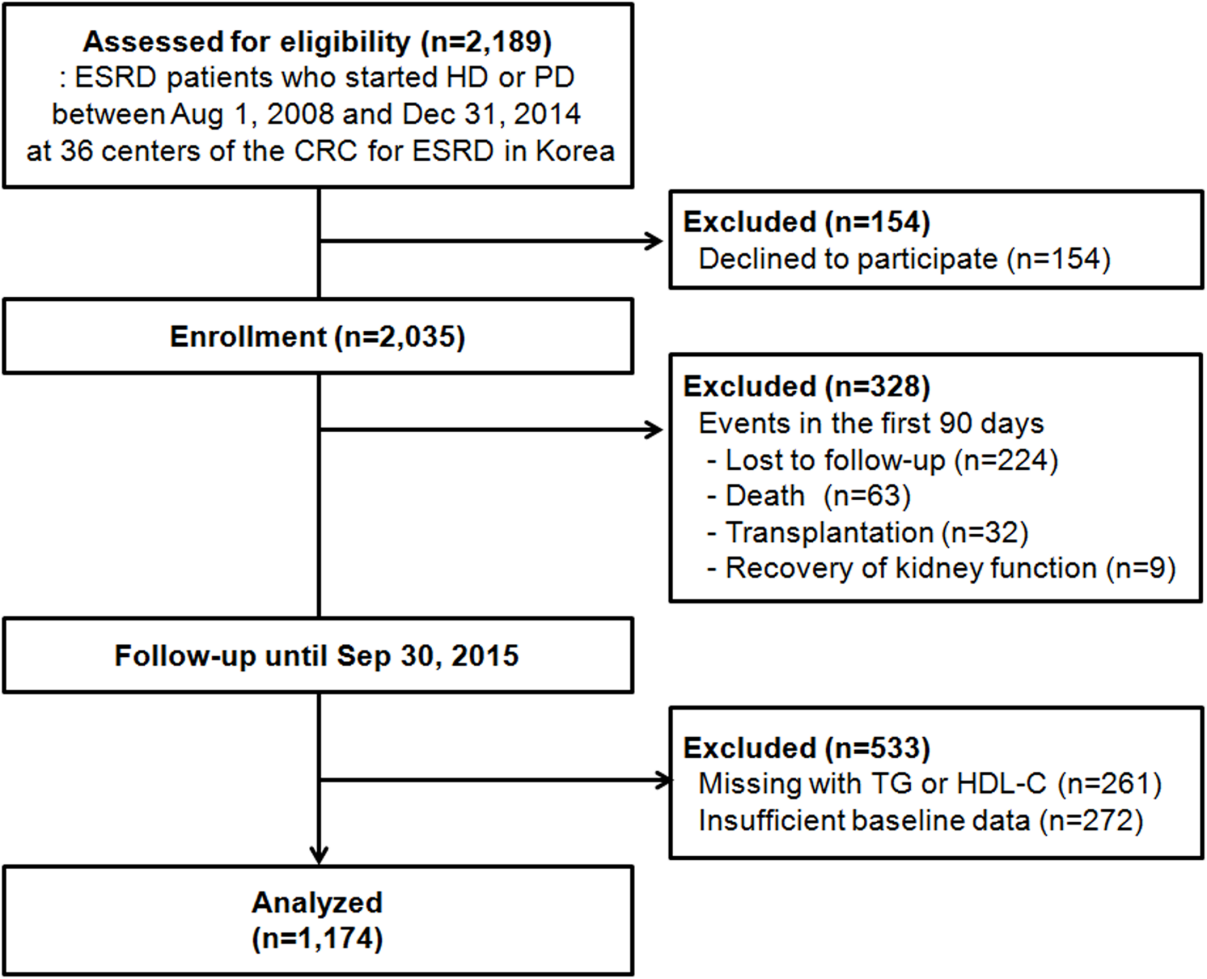
Flow diagram of patients. Among 2,189 incident dialysis patients, 1,015 patients were excluded. A total of 1,174 patients were finally analyzed. *Abbreviations*: CRC, clinical research center; ESRD, end-stage renal disease; HD, hemodialysis; HDL-C, high-density lipoprotein cholesterol; PD, peritoneal dialysis; TG, triglyceride.

All ESRD patients who started HD or PD between August 1, 2008 and December 31, 2014 at 31 centers of the Clinical Research Center for ESRD (CRC for ESRD) in the Republic of Korea were initially screened for this study. This study is part of a nationwide multicenter prospective cohort study of ESRD patients that aimed to improve clinical outcomes and to develop efficient treatment guidelines in the Republic of Korea (clinicaltrial.gov NCT00931970). Patients who refused to participate in the study, who did not maintain dialysis for the first 3 months, who had missing with TG or HDL-C, or who did not have intact parathyroid hormone, high-sensitivity C-reactive protein (hs-CRP), residual kidney function or residual urine output data were excluded from the initially screened 2,189 patients. In total, 1,174 incident dialysis patients were included in the final analyses (Fig 1). Meanwhile, there were no significant differences in baseline characteristics, all-cause mortality, and cardiovascular mortality between 1,174 included and 533 excluded patients, except for the cause of chronic kidney disease (S1 Table). Patients who were excluded had more kidney failure with unknown causes. This study was carried out in accordance with the Declaration of Helsinki. The study protocol was approved by the Institutional Review Board of each participating center (S1 Text for full names), and all patients provided their written informed consent to participate in the study.

### Data collection

Demographic, clinical, and laboratory data were extracted from the CRC for ESRD electronic database. Demographic and clinical data including age, sex, dialysis modality, primary renal disease, comorbidities, body mass index (BMI), and medications were collected at the study enrollment. Laboratory data were measured from fasting blood samples at 3 months after dialysis initiation. AIP was calculated from plasma TG and HDL-C (log [TG/HDL-C]). In HD patients, blood was taken at the predialysis period and a single pooled Kt/V urea was obtained on the day of the midweek dialysis session. In PD patients, blood was taken under usual overnight peritoneal dialysate volume and glucose concentrations, and weekly peritoneal Kt/V urea was calculated using PD Adequest^®^, version 2.0 for Windows software (Baxter Healthcare, Deerfield, IL, USA). Residual kidney function and residual urine volume were determined by timed urine collection, in a 44-hour interdialytic period in HD patients and 24-hour urine collection in PD patients.

### Follow-up and outcomes

Study participants were followed until September 30, 2015. All death events and the cause of death were retrieved from the CRC for ESRD database and collated with the medical records at each participating center. Death events were also confirmed by the Korea National Statistics database. Cardiovascular death was defined as death from myocardial infarction, unstable angina, new onset, or worsening of congestive heart failure, stroke, or arterial revascularization. Loss to follow-up, kidney transplantation, or recovery of renal function was censored at the end of dialysis treatment.

### Statistical analysis

Statistical analyses were performed using SPSS for Windows version 20.0 (IBM Corp., Armonk, NY, USA) and R (R Foundation for Statistical Computing, Vienna, Austria; www.r-project.org). Continuous variables were expressed as the mean ± standard deviation or the median (interquartile range [IQR]) and categorical variables were expressed as a number (percentage). Patients were categorized into five groups according to the AIP quintiles (Q1 to Q5). Baseline characteristics were compared among the groups using ANOVA with the Bonferroni post-hoc test or the Kruskal-Wallis test for continuous variables, and the chi-square test for categorical variables. The association between the AIP and baseline characteristics was examined by uni- and multivariate linear regression analyses. The prognostic value of the AIP was ascertained by Cox proportional hazard regression analyses. Multivariate Cox models were constructed including age, sex, presence of diabetes mellitus, prior history of cardiovascular disease, dialysis modality, BMI, serum albumin levels, hs-CRP levels, and residual urine volume, which were significant risk factors for mortality in the univariate Cox analyses or independent variables associated with the AIP in the linear regression analyses. Sensitivity analyses were done only for all-cause mortality due to the limited number of cardiovascular death events. Because there was interaction between the AIP and BMI for all-cause mortality (P for interaction = 0.03), subgroup analyses were performed. Patients were divided into underweight to normal BMI (BMI <22.9 kg/m^2^) and overweight to obese (BMI ≥23.0 kg/m^2^) groups using the World Health Organization recommendations for Asian populations [18]. Although there was no interaction between the AIP and diabetes mellitus (P for interaction = 0.12) or dialysis modality (P for interaction = 0.62), stratified analyses were performed due to clinical relevance. P <0.05 was considered statistically significant.

## Results

### Baseline characteristics according to the AIP categories

Baseline demographics, clinical characteristics, and laboratory data according to the AIP categories are shown in Table 1 and Table 2. The mean age was 55.4±14.2 years, and 721 patients (61.4%) were men. In total, 740 HD and 434 PD patients were included in this study. The median value of the AIP was 0.47 (IQR, 0.26–0.66) for all patients, and the median value of the AIP for each quintile was 0.06, 0.31, 0.47, 0.62, and 0.84, respectively. In the patients, there was a significant increase of diabetes, BMI, serum glucose, total cholesterol, and low-density lipoprotein cholesterol (LDL-C) concentrations as the AIP increased.

**Table 1:** Baseline demographics and clinical characteristics of subjects according to the AIP categories.

	All	Quintile 1	Quintile 2	Quintile 3	Quintile 4	Quintile 5	
	(min, max)	(-0.57, 0.20)	(0.21, 0.39)	(0.40, 0.54)	(0.55, 0.70)	(0.71, 1.58)	
	(n = 1,174)	(n = 234)	(n = 236)	(n = 235)	(n = 235)	(n = 234)	P
**AIP**	0.47 (0.26–0.66)	0.06 (-0.58–0.14)	0.31 (0.26–0.36)	0.47 (0.42–0.51)	0.62 (0.58–0.66)	0.84 (0.77–0.95)	<0.001
**Age, years**	55.4 ± 14.2	55.1 ± 15.0	57.0 ± 14.0	54.5 ± 14.6	55.8 ± 13.3	54.4 ± 14.0	0.24
**Men, n (%)**	721 (61.4%)	151 (64.5%)	142 (60.2%)	133 (56.6%)	144 (61.3%)	151 (64.5%)	0.36
**Hemodialysis, n (%)**	740 (63.0%)	158 (67.5%)	153 (64.8%)	147 (62.6%)	140 (58.6%)	142 (60.7%)	0.39
**Primary renal disease, n (%)**							0.33
Diabetic nephropathy	599 (51.0%)	108 (46.2%)	109 (46.2%)	117 (49.8%)	128 (54.5%)	137 (58.5%)	
Hypertensive nephrosclerosis	209 (17.8%)	47 (20.1%)	49 (20.8%)	43 (18.3%)	35 (14.9%)	35 (15.0%)	
Glomerulonephritis	168 (14.3%)	38 (16.2%)	30 (12.7%)	39 (16.6%)	34 (14.5%)	27 (11.5%)	
Polycystic kidney disease	23 (2.0%)	7 (3.0%)	6 (2.5%)	5 (2.1%)	3 (1.3%)	2 (0.9%)	
^a^Others	38 (3.2%)	4 (1.7%)	10 (4.2%)	9 (3.8%)	6 (2.6%)	9 (3.8%)	
Unknown	137 (11.7%)	30 (12.8%)	32 (13.6%)	22 (9.4%)	29 (12.3%)	24 (10.3%)	
**Comorbid disease, n (%)**							
Diabetes mellitus	661 (55.3%)	116 (49.6%)	127 (53.8%)	127 (54.0%)	141 (60.0%)	150 (64.1%)	0.01
CAD	160 (13.6%)	29 (12.4%)	26 (11.0%)	34 (14.5%)	37 (15.7%)	34 (14.5%)	0.58
PAD	97 (8.3%)	23 (9.8%)	16 (6.8%)	19 (8.1%)	19 (8.1%)	20 (8.5%)	0.83
CVA	94 (8.0%)	18 (7.7%)	21 (8.9%)	16 (6.8%)	21 (8.9%)	18 (7.7%)	0.9
CHF	160 (13.6%)	25 (10.7%)	34 (14.4%)	33 (14.0%)	35 (14.9%)	33 (14.1%)	0.69
^b^CVD	373 (31.8%)	74 (31.6%)	68 (28.8%)	77 (32.8%)	81 (34.5%)	73 (31.2%)	0.76
**Smoker, n (%)**	544 (46.3%)	104 (44.4%)	113 (47.9%)	97 (41.3%)	107 (45.5%)	123 (52.6%)	0.15
**SBP, mmHg**	141.8 ± 22.5	142.9 ± 23.0	143.3 ± 22.9	141.1 ± 22.2	140.3 ± 21.3	141.2 ± 22.8	0.54
**DBP, mmHg**	78.2 ± 13.7	78.7 ± 14.6	78.1 ± 13.8	77.8 ± 12.9	77.8 ± 13.7	78.6 ± 13.7	0.9
**BMI, kg/m**^**2**^	22.9 ± 3.3	21.6 ± 2.9	22.4 ± 3.0	22.9 ± 3.0	23.4 ± 3.6	24.1 ± 3.5	<0.001
**Medications, n (%)**							
RAS blockers	712 (60.6%)	143 (61.1%)	149 (63.1%)	145 (61.7%)	137 (58.3%)	138 (59.0%)	0.82
Beta-blockers	615 (52.4%)	117 (50.0%)	124 (52.5%)	115 (48.9%)	133 (56.6%)	126 (53.8%)	0.47
Calcium channel blockers	746 (63.5%)	146 (62.4%)	158 (66.9%)	154 (65.5%)	135 (57.4%)	153 (65.4%)	0.21
Diuretics	681 (58.0%)	136 (58.1%)	140 (59.3%)	128 (54.5%)	141 (60.0%)	136 (50.4%)	0.78

*Note*: Data are expressed as the mean ± standard deviation, median (interquartile range), or number of patients (percent).

^a^Others: Interstitial nephritis, obstructive uropathy, and post status of nephrectomy

^b^CVD: A composite of CAD, PAD, CVA, and CHF

*Abbreviations*: AIP, atherogenic index of plasma; BMI, body mass index; CAD, coronary artery disease; CHF, congestive heart failure; CVD, cardiovascular disease; CVA, cerebrovascular accidents; DBP, diastolic blood pressure; PAD, peripheral artery disease; RAS, renin-angiotensin system; SBP, systolic blood pressure.

**Table 2:** Laboratory data at 3 months after dialysis initiation according to the AIP categories.

	All	Quintile 1	Quintile 2	Quintile 3	Quintile 4	Quintile 5	
	(min, max)	(-0.57, 0.20)	(0.21, 0.39)	(0.40, 0.54)	(0.55, 0.70)	(0.71, 1.58)	
	(n = 1,174)	(n = 234)	(n = 236)	(n = 235)	(n = 235)	(n = 234)	P
**AIP**	0.47 (0.26–0.66)	0.06 (-0.58–0.14)	0.31 (0.26–0.36)	0.47 (0.42–0.51)	0.62 (0.58–0.66)	0.84 (0.77–0.95)	<0.001
**Hemoglobin, g/L**	88 ± 16	88 ± 16	88 ± 17	88 ± 18	88 ± 16	89 ± 16	0.85
**BUN, mmol/L**	29.8 ± 13.4	30.6 ± 13.1	29.3 ± 14.0	30.1 ± 13.5	29.3 ± 12.1	29.6 ± 14.1	0.80
**Creatinine, μmol/L**	769 ± 362	769 ± 362	743 ± 309	796 ± 371	778 ± 371	778 ± 380	0.49
**Albumin, g/L**	33 ± 6	33 ± 6	33 ± 6	34 ± 6	33 ± 6	36 ± 6	0.62
**Glucose, mmol/L**	7.7 ± 4.1	7.3 ± 3.6	7.3 ± 3.8	7.4 ± 3.8	8.3 ± 4.6	8.2 ± 4.5	0.01
**TG, mmol/L**	3.3 ± 2.1	1.5 ± 0.5	2.3 ± 0.6	3.0 ± 0.8	3.7 ± 0.9	5.9 ± 2.8	<0.001
**Total cholesterol, mmol/L**	4.1 ± 1.3	3.8 ± 1.0	4.0 ± 1.1	4.0 ± 1.1	4.2 ± 1.3	4.6 ± 1.7	<0.001
**LDL-C, mmol/L**	2.3 ± 1.0	2.0 ± 0.7	2.3 ± 0.9	2.3 ± 0.9	2.5 ± 1.0	2.5 ± 1.3	<0.001
**HDL-C, mmol/L**	1.0 ± 0.4	1.4 ± 0.4	1.2 ± 0.3	1.0 ± 0.3	0.9 ± 0.2	0.7 ± 0.2	<0.001
**Calcium, mmol/L**	2.0 ± 0.3	1.9 ± 0.3	1.9 ± 0.3	1.9 ± 0.3	2.0 ± 0.3	2.0 ± 0.3	0.79
**Phosphorus, mmol/L**	1.8 ± 0.6	1.8 ± 0.7	1.8 ± 0.6	1.8 ± 0.6	1.8 ± 0.7	1.8 ± 0.6	0.77
**iPTH, ng/L**	215 (123–353)	227 (124–367)	205 (122–354)	210 (124–320)	196 (127–320)	200 (117–325)	0.43
**hs-CRP, mg/L**	2.6 (0.7–10.3)	2.6 (0.6–9.4)	2.6 (0.6–12.7)	2.5 (0.6–9.5)	2.9 (0.8–9.2)	3.1 (0.9–12.5)	0.20
^**a**^**Dialysis Kt/V urea (HD)**	1.4 ± 0.4	1.4 ± 0.4	1.4 ± 0.3	1.4 ± 0.3	1.4 ± 0.4	1.4 ± 0.5	0.42
^**b**^**Weekly Kt/V urea (PD)**	1.4 ± 0.5	1.4 ± 0.5	1.4 ± 0.5	1.4 ± 0.5	1.4 ± 0.5	1.4 ± 0.6	0.75
**RKF, mL/min/1.73m**^**2**^	5.5 ± 3.7	5.7 ± 4.7	5.6 ± 3.3	5.1 ± 3.1	5.4 ± 2.9	5.5 ± 4.3	0.49
**Urine volume, L/day**	1.1 ± 0.7	1.0 ± 0.6	1.1 ± 0.7	1.0 ± 0.6	1.0 ± 0.6	1.2 ± 0.8	0.11

*Note*: Data are expressed as mean ± standard deviation or median (interquartile range).

^a^Dialysis Kt/V urea were meausred in 740 HD patients.

^b^Weekly peritoneal Kt/V urea were measured in 434 PD patients.

*Abbreviations*: AIP, atherogenic index of plasma; BUN, blood urea nitrogen; HD, hemodialysis; HDL-C, high-density lipoprotein cholesterol; hs-CRP, high-sensitivity C-reactive protein; iPTH, intact parathyroid hormone; LDL-C, low density lipoprotein cholesterol; PD, peritoneal dialysis; RKF, residual kidney function; TG, triglyceride.

### Association between AIP and baseline characteristics

In univariate linear regression analyses, the BMI and log hs-CRP levels were positively associated with the AIP. The AIP was significantly higher in PD patients and diabetic patients. Multivariate linear regression analyses indicated that a higher AIP was independently associated with PD, diabetes mellitus, a higher BMI, and log hs-CRP concentrations (Table 3).

**Table 3:** Uni- and multivariate linear regression analysis of clinical and biochemical variables for higher AIP.

	Univariate		^a^Multivariate	
	β (95% CI)	P	β (95% CI)	P
**Age (per year)**	0.001 (-0.002, 0.001)	0.47	-0.001 (-0.002, 0.001)	0.14
**Women (vs. men)**	0.001 (-0.036, 0.036)	0.9	0.015 (-0.020, 0.050)	0.40
**PD (vs. HD)**	0.046 (0.010, 0.082)	0.01	0.057 (0.022, 0.093)	0.002
**DM (vs. no)**	0.053 (0.017, 0.088)	0.003	0.044 (0.008, 0.0794)	0.02
**BMI (per kg/m**^**2**^**)**	0.024 (0.019, 0.029)	<0.001	0.024 (0.018, 0.029)	<0.001
**log hs-CRP (per mg/L)**	0.022 (0.001, 0.043)	0.04	0.026 (0.004, 0.047)	0.02

^a^Adjusted for age, sex, dialysis modality, DM, BMI, and log hs-CRP levels

*Abbreviations*: AIP, atherogenic index of plasma; BMI, body mass index; CI, confidence interval; DM, diabetes mellitus; HD, hemodialysis; hs-CRP, high-sensitivity C-reactive protein; PD, peritoneal dialysis.

### Independent prognostic value of AIP for mortality

During a mean follow-up duration of 33.2±18.2 months, 170 patients (14.5%) died, and cardiovascular death was observed in 55 patients (4.7%) (Table 4). Incidence rates of all-cause death and cardiovascular death were the lowest (21 patients, 8.9%; 7 patients, 3.0%, respectively) in patients in the AIP quintile 3 and the highest (40 patients, 17.1%; 16 patients, 6.8%, respectively) in patients in the AIP quintile 5 group.

**Table 4:** All-cause and cardiovascular death events according to the AIP categories.

	All	Quintile 1	Quintile 2	Quintile 3	Quintile 4	Quintile 5
	(n = 1,174)	(n = 234)	(n = 236)	(n = 235)	(n = 235)	(n = 234)
**Follow-up duration (month)**	33.2 ± 18.2	34.5 ± 17.4	32.8 ± 17.9	31.6 ± 18.9	35.3 ± 18.2	31.9 ± 18.6
**All-cause death, n (%)**	170 (14.5%)	36 (15.4%)	37 (15.7%)	21 (8.9%)	36 (15.3%)	40 (17.1%)
**Cardiovascular death, n (%)**	55 (4.7%)	11 (4.7%)	9 (3.8%)	7 (3.0%)	12 (5.1%)	16 (6.8%)

*Abbreviations*: AIP, atherogenic index of plasma.

**Fig 2:**
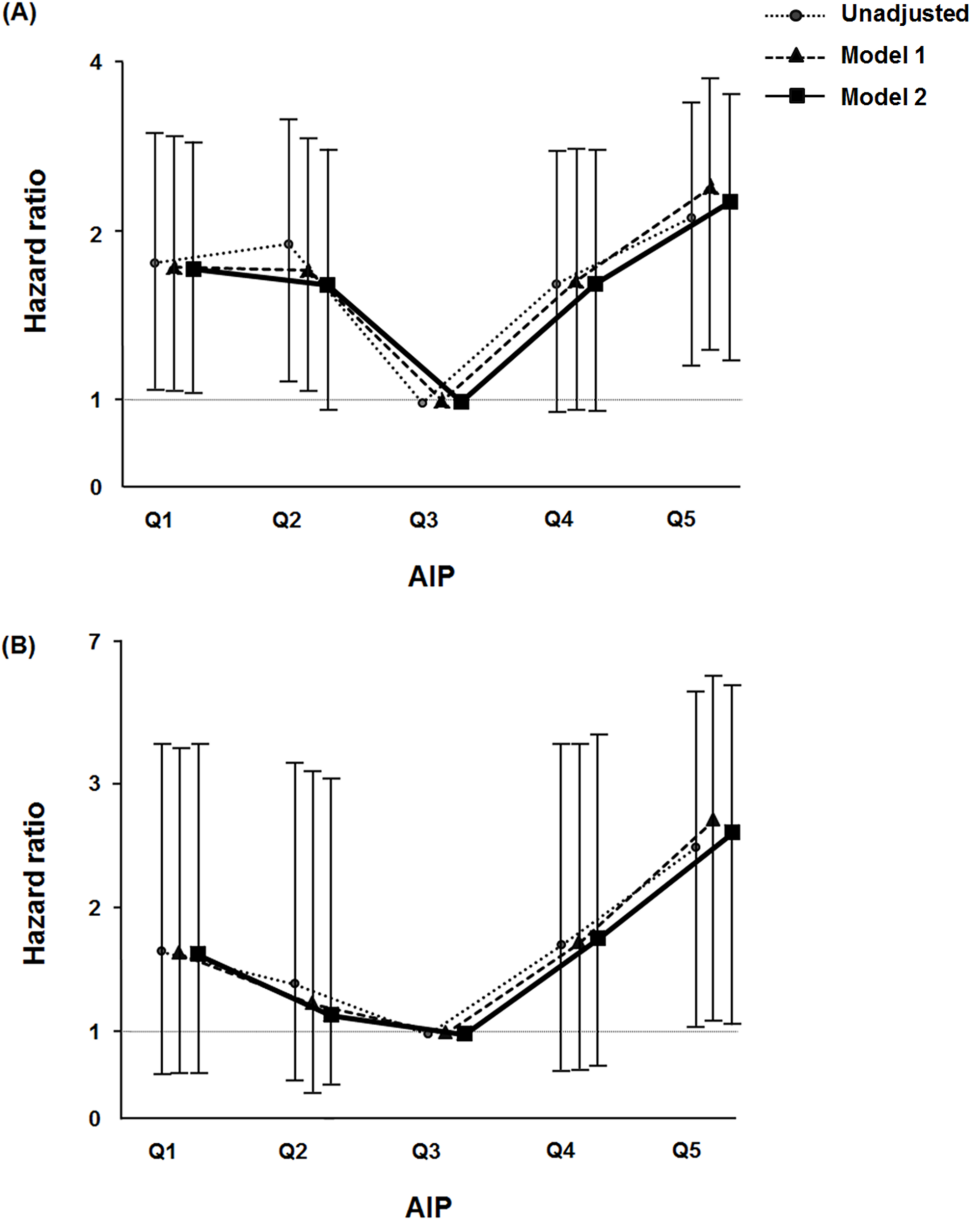
Hazard ratios of all-cause and cardiovascular mortality for AIP categories in 1,174 incident dialysis patients. (A) all-cause mortality and (B) cardiovascular mortality. Model 1: adjusted for sex, age, diabetes mellitus, previous history of cardiovascular disease, dialysis modality, and body mass index; Model 2: Model 1+serum albumin, log hs-CRP, and residual urine volume. *Abbreviations*: AIP, atherogenic index of plasma; hs-CRP, high-sensitivity C-reactive protein.

To determine the independent prognostic value of the AIP for all-cause and cardiovascular mortality, Cox’s regression analyses were performed (Fig 2). Multivariate Cox analyses demonstrated that the lowest (Q1) and the highest (Q5) AIP groups were independently associated with higher all-cause mortality compared with patients of quintile 3 (reference group). The hazard ratios (HRs) of each AIP group for all-cause mortality were 1.76 (Q1, 95% confidence interval [95% CI] = 1.02–3.03), 1.67 (Q2, 95% CI = 0.97–2.86), 1.68 (Q4, 95% CI = 0.98–2.89), and 2.15 (Q5, 95% CI = 1.26–3.65), respectively. In terms of cardiovascular mortality, only the highest AIP group (Q5, HR = 2.59, 95% CI = 1.06–6.34) was significantly associated with increased risk of cardiovascular mortality. The other groups did not show a significant relationship with cardiovascular mortality (Q1, HR = 1.63, 95% CI = 0.63–4.26; Q2, HR = 1.15, 95% CI = 0.43–3.12; Q4, HR = 1.75, 95% CI = 0.69–4.47; Q3 as reference group). Furthermore, additional adjustment of serum calcium and phosphorus did not change the main results. The lowest (Q1, HR = 1.79, 95% CI = 1.03–3.11) and the highest (Q5, HR = 2.00, 95% CI = 1.17–3.43) AIP groups were significantly associated with greater risk of all-cause mortality compared with the reference group (Q3). The highest AIP group (Q5, HR = 2.55, 95% CI = 1.03–6.30) showed a higher risk of cardiovascular mortality (S2 Table).

**Fig 3:**
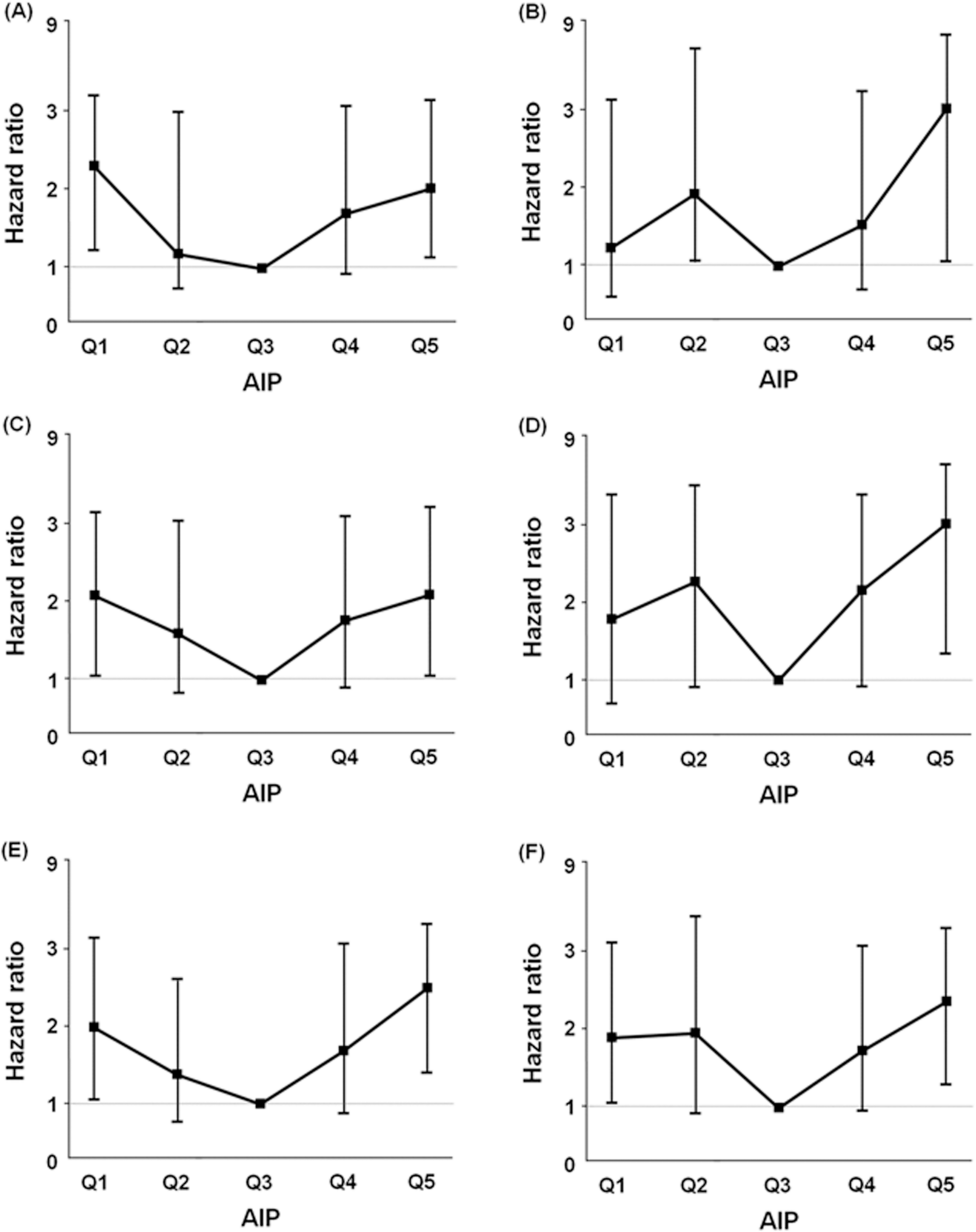
Adjusted hazard ratios of all-cause mortality for AIP categories in different subgroups. (A) in 513 non-diabetic, (B) in 661 diabetic patients, (C) in 653 underweight to normal BMI patients, (D) in 521 overweight to obese patients, (E) in 740 HD, and (F) in 434 PD patients. Adjusted for age, sex, dialysis modality, modified Charlson comorbidity index, BMI, serum albumin, log hs-CRP, and residual urine volume. In subgroup analyses regarding HD and PD, dialysis modality was not adjusted. *Abbreviations*: AIP, atherogenic index of plasma; BMI, body mass index; hs-CRP, high-sensitivity C-reactive protein.

For sensitivity analyses, the independent prognostic value of the AIP for all-cause mortality was tested in several subgroups (Fig 3). The prognostic value of the highest quintile of the AIP (Q5) for all-cause mortality was significant in both diabetic (HR = 2.90, 95% CI = 1.01–8.32) and non-diabetic (HR = 2.01, 95% CI = 1.07–3.75) patients. However, an increased mortality risk of the lowest AIP groups was significant only in non-diabetic patients (HR = 2.29, 95% CI = 1.21–4.32). Similarly, the highest AIP group was independently associated with increased risk of all-cause mortality in both the underweight to normal BMI (HR = 2.08, 95% CI = 1.01–4.30) and overweight to obese (HR = 2.97, 95% CI = 1.31–6.76) groups. However, a U-shaped association was significant in only the underweight to normal BMI patients (AIP Q1, HR = 2.07, 95% CI = 1.04–4.11). With regard to dialysis modality, a U-shaped association remained significant in both HD and PD patients. The lowest (Q1, HR = 1.97, 95% CI = 1.05–3.70 in HD and HR = 1.88, 95% CI = 1.01–3.50 in PD) and the highest (Q5, HR = 2.47, 95% CI = 1.34–4.56 in HD and HR = 2.35, 95% CI = 1.28–4.30 in PD) AIP groups were significantly associated with greater all-cause mortality compared with patients in quintile 3.

## Discussion

In the present study, we investigated the prognostic value of the AIP on all-cause and cardiovascular mortality in incident dialysis patients from a nationwide prospective cohort of ESRD patients. Both the lowest (≤0.20) and the highest (≥0.71) AIP groups were independently associated with all-cause mortality risk, showing a U-shaped association. In terms of cardiovascular mortality, only the highest AIP group (≥0.71) showed a significant risk factor. A U-shaped association between the AIP and all-cause mortality was significant in non-diabetic and underweight to normal BMI patients. These findings suggested that the prognostic impact of the AIP was not straightforward.

The AIP has been shown to be a good marker of atherogenicity [7, 19] and implicated with adverse clinical outcomes in the general population [2] and various populations with high cardiovascular risk [3–6, 12, 13]. The prognostic impact of concurrence of elevated TG and low HDL-C was first investigated in the general population [2]. Patients in the highest quintile of the TG/HDL-C ratio had a 16-fold increased risk of myocardial infarction compared with the lowest quintile [2]. Furthermore, a higher TG/HDL-C ratio was also a strong independent predictor of all-cause and cardiovascular mortality in female patients referred for clinically indicated coronary angiography [3]. In patients with prior history of stroke or transient ischemic attacks receiving statin therapy, major cardiovascular events were higher in subjects with atherogenic dyslipidemia, defined as high TG (≥ 150 mg/dL) and low HDL-C (≤ 40 mg/dL), suggesting a significant relationship of atherogenic dyslipidemia with residual cardiovascular risk [5]. To date, in ESRD patients, there have been two single-center studies evaluating the prognostic impact of the TG/HDL-C ratio [12, 13]. Wu et al. [13] investigated the effect of the TG/HDL-C ratio on all-cause and cardiovascular mortality in a retrospective cohort of 1,170 PD patients. In their study, the highest quintile of the TG/HDL-C ratio (≥4.19) showed a significant risk factor for cardiovascular and all-cause mortality. Another study also showed that patients with higher TG/HDL-C ratios (>6.6) had a higher incidence of cardiovascular events, cardiovascular death, and all-cause death in 602 dialysis patients [12]. In the present study, we demonstrated that the highest quintile of the AIP (≥0.71), which is 5.12 for TG/HDL-C ratio, was significantly associated with a 2.15-fold increase in all-cause mortality and 2.59-fold increase in cardiovascular mortality, even after the adjustment of confounding variables.

Although the exact mechanism by which a higher AIP affects increased risk of all-cause and cardiovascular mortality remains unclear, the association of AIP with LDL-C particle size [7], insulin resistance [8–10], and metabolic syndrome [11] can be possible explanations. In a study of 1,433 subjects from 35 cohorts with various risks of atherosclerosis such as hypertension, type 2 diabetes, and dylipidemia, and patients with positive or negative angiography findings, there was a strong positive correlation between AIP and cholesterol esterification rates in apoB-lipoprotein-depleted plasma, an indirect measure of LDL-C particle size [7]. This finding suggested that the AIP directly correlated with the risk of atherosclerosis and that the AIP could be used as a marker of plasma atherogenecity. Another possible explantation for increased cardiovascular risk of a higher AIP could be the association with insulin resistance and metabolic syndrome. The ratio of plasma TG/HDL-C was the best predictor of insulin resistance, which was determined by the steady-state plasma glucose concentration during the insulin suppression test [8]. Insulin resistance increased the concentrations of TG and HDL-C, and in turn, the AIP might affect insulin secretion, β-cell dysfunction, and cause poor glycemic control in type 2 diabetes patients [9, 10]. Moreover, a high AIP was associated with obesity and predicted the incidence of type 2 diabetes, hypertension, and metabolic syndrome [11]. Unfortunately, LDL-C particle size and insulin resistance were not measured in our study, making it difficult to clarify the underlying mechanism for increased cardiovascular risk of a high AIP in ESRD patients on dialysis. However, a cross-sectional evaluation of the AIP and surrogates of cardiovascular disease in dialysis patients supported our findings [19, 20]. In a study of 31 HD patients and 31 healthy volunteers, the AIP and carotid intima medial thickness were higher in HD patients compared with controls [19]. There was a significant positive correlation between the AIP and carotid intima medial thickness in HD patients. Another study also showed that the AIP was significantly associated with increased epicardial adipose tissue, the true visceral fat deposit of the heart in ESRD patients [20]. We did not perform carotid artery ultrasonography or cardiac computed tomography in the current study, but a further study with these cardiovascular surrogates may be helpful to verify a significant association between the AIP and cardiovascular disease in ESRD patients.

Another main finding of this study was a U-shaped association between the AIP and all-cause mortality in incident dialysis patients. Patients in the quintile 3 group of the AIP showed the lowest incidence rate of death events, and the lowest and the highest quintiles of the AIP were significantly associated with higher all-cause mortality risk, compared with patients in the quintile 3 group. Although the reason for higher all-cause mortality risk in the lowest AIP group was not clear, it can be speculated from the deleterious effects of low TG and high HDL-C in dialysis patients. In a large cohort of HD patients, the best survival was observed with moderately high levels (200–249 mg/dL) of TG, suggesting a higher mortality risk with lower levels of TG [15]. This paradoxical higher mortality risk in the low TG group was explained by the effect of malnutrition and inflammation, which are important risk factors for mortality in ESRD patients. In our study, the HRs of the quintile 1 and quintile 2 groups were attenuated after adjustment of serum albumin and hs-CRP concentrations, suggesting a possible association of low TG with malnutrition and inflammation and with mortality. Moreover, emerging evidence indicating that HDL-C may not only be dysfunctional but may also have a harmful effect by promoting inflammation in ESRD patients could be another explanation [16, 21–25]. In a study of 12 stable HD patients and age-matched controls, monocyte anti-chemotactic activity was significantly lower in ESRD patients than in controls, suggesting that HDL-C has a deficient anti-inflammatory property in ESRD patients [21. Yamamoto et al. [24] demonstrated that HDL-C from ESRD patients had reduced anti-chemotactic activity. Furthermore, in vitro activated macrophages exposed to HDL-C of ESRD patients showed an increased cytokine response (tumor necrosis factor-α, interleukin-6, and interleukin-1β) [24]. To investigate this pro-inflammatory property of HDL-C in ESRD patients, Weichhart et al. [23] performed shotgun proteomics analyses and identified 49 HDL-associated proteins in a uremia-specific pattern from HDL-C of HD patients. Of note, serum amyloid A mimicked HDL-C of ESRD patients by promoting inflammatory cytokines, suggesting the role of specific changes in molecular composition on pro-inflammatory property of HDL-C in ESRD patients [23]. Moreover, the combination of high oxidized HDL-C and plasma interleukin-6 was significantly associated with increased carotid intima medial thickness and cardiovascular mortality in prevalent HD patients [22]. Based on these results, we proposed that the deleterious effect of HDL-C could explain the higher risk of mortality in the lowest AIP group. Unfortunately, measurements of chemotactic activity, pro-inflammatory cytokine release from macrophage, or oxidized isoform of HDL-C were not available in our study, so we were unable to confirm our proposal.

Our sensitivity analyses demonstrated that higher all-cause mortality risk in the lowest quintile of the AIP remained significant in non-diabetic patients and underweight to normal BMI patients. Underweight patients had higher risk of mortality compared with overweight-to-obese patients due to the adverse effects of nontraditional risk factors such as malnutrition and inflammation. Therefore, we surmised that the combined risk of low AIP and underweight-to-normal BMI via malnutrition and inflammation might lead to an amplification of the mortality risk for the lowest AIP groups of patients. By contrast, the protective effect of high BMI in overweight-to-obese patients might mitigate the harmful effect of the lowest AIP group, resulting in no significant association with increased mortality risk in these populations. In terms of diabetes, the higher proportion of underweight-to-normal BMI patients in the non-diabetic group could be a possible explanation for the increased mortality risk of the lowest AIP group. In fact, the proportion of underweight-to-normal BMI was significantly higher in non-diabetic patients than in diabetic patients (318 patients [62.0%] vs. 335 patients [50.7%], P<0.001). In addition, a study by Postorino et al. [26] demonstrated that abdominal obesity showed a modifying effect on the association between TG and mortality in HD patients. In patients without abdominal obesity, a fixed (50 mg/dL) excess of TG was associated with a progressively lower risk of mortality, but was associated with a higher risk of mortality in patients with abdominal obesity. Although we did not examine abdominal obesity in this study, we hypothesize that a modifying effect of obesity could be another possible explanation for sensitivity analysis.

This study has several limitations. First, the AIP was not serially determined and we used only baseline values of AIP. We suggest that the prognostic effects of changes in the AIP would be worth investigating. Second, this study was not intended to evaluate the effect of lipid-lowering medications on mortality, and the data concerning lipid-lowering therapy were not available. Although the potential confounding effects of lipid-lowering medications on mortality cannot be excluded, considering the results of recent clinical trials regarding lipid-lowering therapy [27–29], we suggest that these data may not significantly alter the main findings of our study. Third, although there was no significant difference in the baseline characteristics, all-cause mortality, and cardiovascular mortality between included and excluded patients, 1,174 patients may not be representative of the initially enrolled population, resulting in potential selection bias. In this study, 261 patients who had missing with TG or HDL-C were excluded from the final analysis. This study was conducted as an exploratory study to identify risk factors for adverse clinical outcomes; therefore, AIP was not a pre-specified variable. Patients who did not have intact parathyroid hormone, hs-CRP, residual kidney function, or residual urine output data were also excluded. Although many previous studies using nationwide data did not include information regarding hs-CRP or residual kidney function, we excluded patients who lacked measurements of these variables due to their significant effects on clinical outcomes of dialysis patients. These factors can explain the reasons for the large number of missing laboratory values. Meanwhile, the mean age of patients was relatively young and proportion of patients treated with PD was high in the present study. According to a report of the Korean Society of Nephrology [30], the mean age of all dialysis patients, including prevalent and incident patients, was 56.7 years in 2008. The mean age steadily increased to 60.3 years in 2014. In 2014, the mean age of HD patients was 61.1 years, whereas the mean age of PD patients was 55.4 years. Even though we did not select or focus on a young age group, we surmise that the study period (from 2008 to 2012), participants (only incident dialysis patients), and high proportion of PD contributed to the relatively young age of the study subjects. In our study, patients were more likely to be treated with PD as an initial dialysis modality. Because the majority of study participating centers were university hospitals, we postulate that structured pre-dialysis education program, experienced nephrologists familiar with PD, and adequate medical resources for PD may contribute to higher selection for PD. In addition, HD patients were more likely to be transferred to private dialysis units; therefore, the proportion of excluded patients due to follow-up loss within the first 90 days was lower in PD patients than in HD patients (among 224 patients, 204 patients [91.1%] in HD vs. 20 patients [8.9%] in PD, P<0.001). Although subgroup analysis according to dialysis modality did not change the main results, the high proportion of PD patients could lead to potential selection bias. Fourth, by the virtue of the observational study design, this study did not include any information regarding intervention to reduce mortality. Fifth, because only Korean incident dialysis patients were included, the association between AIP and mortality may not be generalized to other ethnic groups or races. Lastly, we did not fully investigate the association between the AIP and cardiovascular mortality via sensitivity analyses due to the small number of cardiovascular deaths. A future study is needed to clarify this issue. Notwithstanding these limitations, our study was the first investigation to determine the independent prognostic value of the AIP in a nationwide prospective dialysis cohort.

## Conclusions

The current study demonstrated that both the highest and the lowest AIP groups were significantly associated with increased risk of all-cause mortality, showing a U-shaped association in incident dialysis patients. Furthermore, a U-shaped association between the AIP and all-cause mortality was significant in non-diabetic and underweight to normal BMI patients. These findings suggest that further studies are needed to identify targets and subgroups that can benefit from interventions of the AIP in ESRD patients.

## Supporting information

S1 Text Thirty-one centers participating in clinical research center for end stage renal disease. (PDF)

S1 Table Comparison of baseline characteristics and outcomes between finally analyzed patients (n = 1,174) and excluded patients (n = 533). (PDF)

S2 Table Multivariate Cox regression analysis of AIP categories for all-cause and cardiovascular mortality including adjustment of serum calcium and phosphorus concentrations. (PDF)
