# Supplementary material for: The atherogenic index of plasma and the risk of mortality in incident dialysis patients: Results from a nationwide prospective cohort in Korea
Source: PLoS One. 2017 May 26;12(5):e0177499. doi: 10.1371/journal.pone.0177499 (PMC5446226; doi:10.1371/journal.pone.0177499)
Supplement: S1 Text — (PDF) [file pone.0177499.s001.pdf]

S1 Text. Thiry-one centers participating in Clinical Research Center for End Stage Renal Disease.

The Catholic University of Korea, Bucheon St. Mary's Hospital; The Catholic University of Korea, Incheon St. Mary's Hospital; The Catholic University of Korea, Seoul St. Mary's Hospital; The Catholic University of Korea, St. Mary's Hospital; The Catholic University of Korea, St. Vincent's Hospital; The Catholic University of Korea, Uijeongbu St. Mary's Hospital; Cheju Halla General Hospital; Chonbuk National University Hospital; Chonnam National University Hospital; Chung-Ang University Medical Center; Chungbuk National University Hospital; Chungnam National University Hospital; Dong-A University Medical Center; Ewha Womans University Medical Center; Fatima Hospital; Gachon Medical School Gil Medical Center; Inje University Busan Paik Hospital; Kyungpook National University Hospital; Kwandong University College of Medicine, Myongji Hospital; National Health Insurance Corporation Ilsan Hospital; National Medical Center; Busan National University Hospital; Samsung Medical Center; Seoul National University Boramae Medical Center; Seoul National University Hospital; Seoul National University, Bundang Hospital; Yeungnam University Medical Center; Yonsei University, Severance Hospital; Yonsei University, Gangnam Severance Hospital; Ulsan University Hospital; Wonju Christian Hospital (in alphabetical order).
