# Supplementary material for: The atherogenic index of plasma and the risk of mortality in incident dialysis patients: Results from a nationwide prospective cohort in Korea
Source: PLoS One. 2017 May 26;12(5):e0177499. doi: 10.1371/journal.pone.0177499 (PMC5446226; doi:10.1371/journal.pone.0177499)
Supplement: S1 Table — (PDF) [file pone.0177499.s002.pdf]

S1 Table. Comparison of baseline characteristics and outcomes between finally analyzed patients (n=1,174) and excluded patients (n=533)

	Finally analyzed patients (n=1,174)	Excluded patients (n=533)	P
**Age, years**	55.4 ± 14.2	55.1 ± 13.8	0.73
**Men, n (%)**	721 (61.4%)	329 (61.7%)	0.9
**Hemodialysis, n (%)**	740 (63.0%)	342 (64.2%)	0.29
**Primary renal disease, n (%)**			<0.001
Diabetic nephropathy	599 (51.0%)	251 (47.1%)	
Hypertensive nephrosclerosis	209 (17.8%)	61 (11.4%)	
Glomerulonephritis	168 (14.3%)	75 (14.1%)	
Polycystic kidney disease	23 (2.0%)	8 (1.5%)	
[a]Others	38 (3.2%)	10 (1.9%)	
Unknown	137 (11.7%)	128 (24.0%)	
**Comorbid disease, n (%)**			
Diabetes mellitus	661 (55.3%)	275 (51.6%)	0.07
[b]CVD	373 (31.8%)	162 (30.4%)	0.61
**Smoker, n (%)**	544 (46.3%)	239 (44.8%)	0.60
**SBP, mmHg**	141.8 ± 22.5	140.9 ± 20.4	0.89
**DBP, mmHg**	78.2 ± 13.7	79.8 ± 12.5	0.18
**BMI, kg/m^2**	22.9 ± 3.3	23.0 ± 3.3	0.72
**Hemoglobin, g/L**	88 ± 16	89 ± 17	0.15
**BUN, mmol/L**	29.8 ± 13.4	29.5 ± 13.9	0.64
**Creatinine, µmol/L**	769 ± 362	743 ± 327	0.19
**Albumin, g/L**	33 ± 6	34 ± 6	0.33
**Glucose, mmol/L**	7.7 ± 4.1	7.6 ± 4.0	0.43
**Calcium, mmol/L**	2.0 ± 0.3	2.0 ± 0.3	0.18
**Phosphorus, mmol/L**	1.8 ± 0.6	1.8 ± 0.6	0.20

**Follow-up duration, months**	33.2 ± 18.2	33.5 ± 17.2	0.80
**Death, n (%)**	170 (14.5%)	66 (12.4%)	0.26
**Cardiovascular death, n (%)**	55 (4.7%)	24 (4.5%)	0.9

Note: Data are expressed as the mean ± standard deviation or number of patients (percent).

[a]Others: Interstitial nephritis, obstructive uropathy, and post status of nephrectomy

[b]CVD: A composite of coronary artery disease, peripheral artery disease, cerebrovascular accident, and congestive heart failure.

Abbreviations: BMI, body mass index; BUN, blood urea nitrogen; CVD, cardiovascular disease; DBP, diastolic blood pressure; SBP, systolic blood pressure.
