# Supplementary material for: The atherogenic index of plasma and the risk of mortality in incident dialysis patients: Results from a nationwide prospective cohort in Korea
Source: PLoS One. 2017 May 26;12(5):e0177499. doi: 10.1371/journal.pone.0177499 (PMC5446226; doi:10.1371/journal.pone.0177499)
Supplement: S2 Table — (PDF) [file pone.0177499.s003.pdf]

S2 Table. Multivariate Cox regression analysis of AIP categories for all-cause and cardiovascular mortality including adjustment of serum calcium and phosphorus concentrations

**Variable**	**All-cause mortality**	**Cardiovascular mortality**
	**HR (95% CI)**	**HR (95% CI)**
**Age, year**	1.04 (1.03-1.06)	1.05 (1.02-1.07)
**Women (versus men)**	0.85 (0.61-1.19)	0.81 (0.45-1.47)
**Diabetes mellitus**	1.21 (0.85-1.72)	1.90 (0.95-3.81)
[a]**Previous CVD**	1.65 (1.20-2.28)	1.59 (0.90-2.79)
**PD (versus HD)**	0.75 (0.53-1.06)	0.71 (0.38-1.34)
**BMI, per kg/m^2**	0.96 (0.91-1.01)	0.93 (0.85-1.02)
**Albumin, per 0.1 g/L**	0.56 (0.41-0.75)	0.46 (0.27-0.76)
**Log hs-CRP, per mg/L**	1.28 (1.05-1.56)	1.43 (1.01-2.04)
**Residual urine volume, per 0.1 L/d**	0.99 (0.96-1.02)	0.98 (0.94-1.03)
**Calcium**		
<8.5 mg/dl	0.44 (0.23-0.84)	0.37 (0.11-1.33)
8.5 to <9.0 mg/dl	0.56 (0.27-1.16)	0.48 (0.12-1.93)
9.0 to <9.5 mg/dl	Reference	Reference
9.5 to <10.2 mg/dl	1.25 (0.43-3.60)	0.53 (0.05-0.57)
≥10.2 mg/dl	0.45 (0.06-3.60)	-
**Phosphorus**		
<4.5 mg/dl	0.99 (0.99-1.50)	1.48 (0.67-3.27)
4.5 to <5.4 mg/dl	Reference	Reference
5.4 to <6.4 mg/dl	1.27 (0.82-1.98)	2.46 (1.10-5.52)
≥6.4 mg/dl	0.80 (0.49-1.28)	0.91 (0.35-2.39)
**AIP categories**		
Quintile 1	1.79 (1.03-3.11)	1.80 (0.68-4.75)
Quintile 2	1.67 (0.97-2.88)	1.16 (0.43-3.18)
Quintile 3	Reference	Reference
Quintile 4	1.71 (0.99-2.95)	1.91 (0.74-4.91)
Quintile 5	2.00 (1.17-3.43)	2.55 (1.03-6.30)

[a]CVD: A composite of coronary artery disease, peripheral artery disease, cerebrovascular accident, and congestive heart failure.

Abbreviations: AIP, atherogenic index of plasma; BMI, body mass index; CVD, cardiovascular disease; DBP, diastolic blood pressure; HD, hemodialysis; hs-CRP, high-sensitivity C-reactive protein; PD, peritoneal dialysis; SBP, systolic blood pressure.
